# Cytomegalovirus Reactivation in Critically Ill Patients With Acute Necrotizing Pancreatitis

**DOI:** 10.1093/ofid/ofaf438

**Published:** 2025-07-23

**Authors:** Umadri Singh, Mohan Gurjar, Atul Garg, Samir Mohindra, Prabhaker Mishra, Rahul Rahul, Shyam S Yadav, Afzal Azim, Banani Poddar

**Affiliations:** Department of Critical Care Medicine, Sanjay Gandhi Postgraduate Institute of Medical Sciences (SGPGIMS), Lucknow, India; Department of Critical Care Medicine, Sanjay Gandhi Postgraduate Institute of Medical Sciences (SGPGIMS), Lucknow, India; Department of Microbiology, Sanjay Gandhi Postgraduate Institute of Medical Sciences (SGPGIMS), Lucknow, India; Department of Gastroenterology, Sanjay Gandhi Postgraduate Institute of Medical Sciences (SGPGIMS), Lucknow, India; Department of Biostatistics and Health Informatics, Sanjay Gandhi Postgraduate Institute of Medical Sciences (SGPGIMS), Lucknow, India; Department of Surgical Gastroenterology, Sanjay Gandhi Postgraduate Institute of Medical Sciences (SGPGIMS), Lucknow, India; Department of Microbiology, Sanjay Gandhi Postgraduate Institute of Medical Sciences (SGPGIMS), Lucknow, India; Department of Critical Care Medicine, Sanjay Gandhi Postgraduate Institute of Medical Sciences (SGPGIMS), Lucknow, India; Department of Critical Care Medicine, Sanjay Gandhi Postgraduate Institute of Medical Sciences (SGPGIMS), Lucknow, India

**Keywords:** acute necrotizing pancreatitis, cytomegalovirus, infection reactivation, intra-abdominal infection, intensive care units

## Abstract

**Background:**

In recent years, cytomegalovirus (CMV) reactivation has been recognized during the critical illness of apparently nonimmunosuppressed patients, impacting their clinical outcomes. However, no study is available on CMV reactivation among acute necrotizing pancreatitis (ANP) patients.

**Methods:**

In this prospective cohort study of adult ANP patients requiring intensive care unit admission with apparently immunocompetent and CMV seropositive status, clinical samples were analyzed on a weekly basis for CMV copies. CMV reactivation was considered when the viral load exceeded 1000 copies/mL in plasma.

**Results:**

During the study period, 284 samples (plasma 149, drain content 125, and necrotic tissue 10) were analyzed from 41 ANP patients. At enrollment, the median (interquartile range) age was 35 (25–40) years, SOFA score was 7 (6–9), and computed tomography severity index of pancreatitis was 10 (6–10). CMV reactivation was detected in the plasma of 13 patients (31.7%) with an average viral load (range) of 3900 (1500–5600) copies/mL. These patients had higher rates of intra-abdominal (92%) and bloodstream infections (77%). Among these individuals, the maximum CMV in plasma was 8180 (7500–38 500) copies/mL and 3200 (2250–4075) copies/mL (*P* = .01), while in drain samples it was 81 675 (63 750–101 750) copies/mL and 17 250 (6077–41 952) copies/mL (*P* = .02), in nonsurvivors and survivors, respectively. CMV copies (per mL) in the pancreatic necrosum (n = 7) were higher 29 200 (14 700–47 500) compared with concurrent samples of drain (10 000 [4135–31 250]) and plasma (1500 [0–12 955]) from the same patients (*P* = .48).

**Conclusions:**

Up to one-third of ANP patients experienced CMV reactivation. Higher and persistent CMV copies in the clinical samples were associated with poorer clinical outcomes.

**ClinicalTrials.gov Identifier:**

NCT05898048.

In recent years, cytomegalovirus (CMV) reactivation has been recognized during critical illness in apparently nonimmunosuppressed patients. After the first description of histologically proven CMV pneumonia in nonimmunosuppressed patients requiring mechanical ventilation, subsequent evidence reported CMV reactivation incidence as high as 20%–40% among the intensive care unit (ICU) population [1, 2]. Studies from specific cohorts of critically ill patients like sepsis, septic shock, and acute respiratory distress syndrome (ARDS) have demonstrated significantly higher morbidity and mortality in critically ill patients who had CMV reactivation [3–6]. A systematic review of 22 studies by Lachance et al. revealed that the presence of CMV reactivation is associated with longer duration of mechanical ventilation, more nosocomial infections, extended intensive care unit (ICU) stays, and increased overall mortality in immunocompetent critically ill patients [7]. The reactivation of dormant CMV viral infection occurs due to immune paralysis of the host during acute illness by many pathways including sepsis [3, 8–10].

Acute pancreatitis is a systemic disease with a wide spectrum of presentations, ranging from mild cases with favorable outcomes to severe cases often requiring admission to the ICU and associated with high morbidity and mortality [11, 12]. In patients with acute necrotizing pancreatitis (ANP), the necrotic debris becomes infected during their clinical course in more than one-third of cases, worsening the outcome [13, 14]. The most common organisms isolated are gram-negative bacteria, mainly *Escherichia coli, Klebsiella pneumoniae,* and *Pseudomonas aeruginosa,* followed by gram-positive bacterial and fungal infections, in these critically ill patients [13, 15–17].

In general, intra-abdominal infections represent the second leading cause of infectious morbidity and mortality among ICU patients [18]. There is a paucity of data on CMV reactivation among severe pancreatitis patients. For this reason, we aimed to study the prevalence of CMV reactivation and its viral load kinetics in critically ill ANP patients.

## METHODS

### Study Design and Patient Population

This single-center prospective observational study was conducted at a 30-bed ICU in a tertiary care university hospital from June 2023 to November 2024. The primary objective of this study was to evaluate the prevalence of CMV reactivation in ANP patients and its viral load kinetics in clinical samples of blood as well as abdominal drain content and pancreatic necrotic tissue, if available, during the clinical course of their ICU stay. This study was registered on ClinicalTrials.gov (Identifier: NCT05898048).

All adult ICU patients (age >18 years) with ANP with a minimum duration of illness of 2 weeks were screened for CMV-seropositive status (presence of anti-CMV immunoglobulin G [IgG] antibodies). Exclusion criteria were duration of pancreatitis >10 weeks at ICU admission, use of antiviral agents (against any herpesvirus family) within the previous 7 days, known or suspected immunodeficiency (solid organ or stem cell transplantation, HIV infection, hematologic malignancy) or recent administration of immunosuppressive therapies (prednisone >0.1 mg/kg/d for >3 months, or 1 mg/kg/d >3 weeks or an equivalent steroid), recent history of chemotherapy or radiotherapy, pregnancy, life expectancy of <72 hours, and not providing consent for the study.

### Patient Consent

The study protocol was approved by the institutional ethics committee (IEC code: 2023-51-DM-130), and informed consent was collected from the participants or their legal representatives before inclusion in the study.

### Definitions and Sample Follow-up

Acute necrotizing pancreatitis was defined based on clinical signs and symptoms, laboratory parameters, and the presence of nonenhancing areas of the pancreas observed on contrast-enhanced computed tomography (CECT) scans [19]. We screened these patients for CMV seropositive status at the time of enrollment by testing serum for anti-CMV IgG antibodies by enzyme-linked immunosorbent assay, and any subsequent detection of CMV DNA in follow-up samples was considered reactivation of the latent virus, rather than a new primary infection. However, in our study, CMV reactivation was defined as a viral load exceeding 1000 copies/mL in plasma, irrespective of CMV copies in the drain content, in patients with confirmed IgG seropositivity.

Clinical samples from enrolled patients were collected every week, from the third week to the 10th week of illness, during their ICU stay. The reason for choosing this time interval was related to the typical clinical course of severe acute pancreatitis [20]. Blood samples (1.0 mL in EDTA vials) were collected from venous catheters, and abdominal drain content (2.0 mL in a sterile container) was collected from the existing abdominal drain nearest the pancreas, whenever available. If patients underwent necrosectomy, a 1-time pancreatic necrosum was also collected.

### CMV Quantitative Assessment

DNA extraction was performed on 200 μL of clinical specimen using a QIAamp DNA mini kit (Qiagen, Inc., Valencia, CA, USA) as per manufacturer instructions, and the extracted DNA was stored at −80°C for further work. The polymerase chain reaction (PCR) reaction was performed in a total volume of 25 μL (15 μL of PCR reaction mixture including probe and primers, plus 10 μL of template DNA) using *artus* CMV PCR RG Kits (Qiagen, Inc., Valencia, CA, USA). The real-time PCR reactions were carried out on the ABI 7500 Fast system. Setting the thermos-cycling conditions was an initial step of 10 minutes at 95°C and 40 cycles of 30 seconds at 95°C, 30 seconds at 57°C, and 30 seconds at 72°C. Each PCR run included a set of quantitative calibrators corresponding to 2.0–6.0 log_10_ copies/mL. The CMV DNA load is calculated from the standard curve and expressed as the number of CMV DNA copies/mL as per manufacturer instructions (1 IU/mL = 1.64 copies/mL). Laboratory results specific to CMV were not made available to the treating team as currently screening for CMV reactivation is not part of routine clinical practice in the nonimmunosuppressed ICU patient population.

### Sample Size and Statistical Analysis

Among screened patients with ANP, we expected 85%–90% of patients to be seropositive for IgG, as in the general population [21]. Also, from the previous studies, the prevalence of CMV reactivation in nonimmunosuppressed ICU populations was around 40%, which further increased to >50% if the ICU stay was prolonged [2, 5, 6]. We assumed that 50% of seropositive (IgG) patients would develop CMV reactivation during their predefined time period; the study design accommodates for uncertainty and variability in the true prevalence of CMV reactivation in the cohort of severe acute pancreatitis, and the maximum possible sample size could be achieved (as variability is proportional to the sample size). Taking a 15% margin of error in assumed prevalence (ie, expected range of 35% to 65%), with a 2-sided 95% CI, the estimated sample size was 43. Descriptive statistics of the continuous variables are presented as median (interquartile range [IQR]), while categorical variables are presented as frequency (%). The Kolmogorov-Smirnov (K-S) test was used to test the normality of the continuous variables. The data either had a non-normal distribution or close to non-normal, and resultant nonparametric approaches were used to compare the groups. The Mann-Whitney *U* test was used to compare medians, whereas the chi-square test or Fisher exact test was used to compare proportions between the 2 groups. Classification and regression tree (CART) analysis was used to present the distribution or association of the different variables in patients with or without CMV reactivation. A *P* value <.05 was considered statistically significant. Statistical Package for Social Sciences, version 23 (IBM, Chicago, IL, USA), and MedCalc Software were used for data analysis.

## RESULTS

### Participants

**Figure 1.**
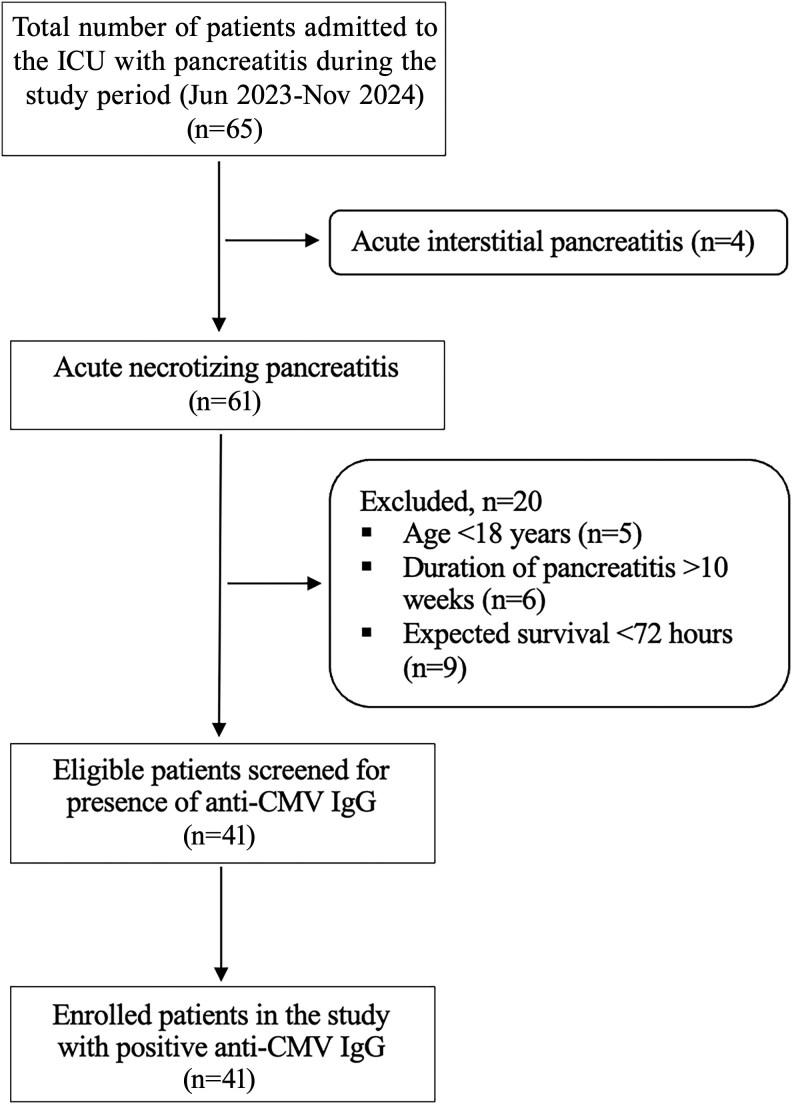
Participant inclusion the study. Abbreviations: CMV, cytomegalovirus; IgG, immunoglobulin G.

During the study period, 65 patients with a diagnosis of pancreatitis were admitted to the ICU, of whom 61 had ANP. As per inclusion and exclusion criteria, 41 patients were screened for the presence of anti-CMV IgG. All these patients were positive for anti-CMV IgG and enrolled in this study (Figure 1). The median (IQR) age was 35 (25–40) years, and 30 (73%) were male. Only 2 patients had a history of comorbidity (diabetes mellitus), owing to a comparatively younger cohort. The main etiology of ANP was biliary (44%), followed by ethanol (27%) related. The median (IQR) severity scores at ICU admission were an Acute Physiology and Chronic Health Evaluation score (APACHE II) of 16 (14–20) and Sequential Organ Failure Assessment (SOFA) score of 7 (6–9) (Table 1).

**Table 1.** Demographics, Clinical Characteristics, and Outcomes of the Study Population (n = 41)

Variables	All Patients (n = 41)	CMV Reactivation Group (n = 13)	CMV Nonreactivation Group (n = 28)	*P* Value
A, At ICU admission				
Age, median (IQR), y	35 (25–40)	35 (22–42)	34.5 (25–40)	.96
Male, No. (%)	30 (73.17)	11 (84.6)	19 (67.86)	.51
ICU severity score				
APACHE II score, median (IQR)	16 (14–20)	14 (1420)	17 (1321)	.71
SOFA score, median (IQR)	7 (6–9)	8 (6–8)	6 (4–9)	.61
Etiology of pancreatitis, No. (%)				
Biliary	18 (43.9)	7 (53.85)	11 (39.2)	
Ethanol	11 (26.83)	4 (30.7)	7 (25)	.58
Post-ERCP	1 (2.44)	0 (0)	1 (3.57)	
Unknown	11 (26.83)	2 (15.35)	9 (32)	
B, At study inclusion				
SOFA score, median (IQR)	7 (6–9)	7 (6,8)	7 (5,9)	.98
Day of illness, median (IQR)	28 (1435)	18 (14–29)	28 (1442)	.17
CT severity index, median (IQR)	10 (6–10)	10 (6–10)	9 (6–10)	.57
Need for MV, No. (%)	19 (46.3)	5 (38.5)	14 (50)	.491
Need for vasopressor (septic shock), No. (%)	30 (73.2)	10 (76.9)	20 (71.4)	.712
Need for RRT, No. (%)	5 (12.2)	2 (15.4)	3 (10.7)	.671
Total leukocyte count, median (IQR), ×10^9^/L	17.9 (15.35–23)	19.1 (17.3–23.7)	16.72 (14.8–22.7)	.272
C, During ICU stay				
Need for MV, No. (%)	33 (80.49)	12 (92.31)	21 (75)	.19
Days of MV, median (IQR)	27 (17–54)	27 (24–56)	20 (15–38)	.15
Need for vasopressor, No. (%)	38 (92.68)	13 (100)	22 (91.6)	.22
Days on vasopressor, median (IQR)	18 (1234)	22 (18–38)	14 (12–26)	.04
Need for RRT, No. (%)	19 (46.34)	8 (61.54)	11 (39.29)	.18
Days on RRT, median (IQR)	0 (0–15)	6 (0–27)	0 (0–3)	.12
Need for intra-abdominal drain, No. (%)	38 (92.7)	12 (92.3)	26 (92.9)	.950
Intra-abdominal infection, No. (%)				
Bacterial	30 (73.17)	12 (92.39)	18 (64.29)	.06
Fungal	9 (21.95)	4 (30.7)	5 (17.86)	.35
BSI, No. (%)				
Bacteremia	24 (58.5)	10 (76.9)	14 (50)	.10
Candidemia	8 (19.5)	5 (38.5)	3 (10.7)	.03
Abdominal complication (noninfectious), No. (%)				
Bleeding	11 (26.8)	4 (30.7)	7 (25)	.69
Perforation	9 (22)	1 (7.7)	8 (28.6)	.13
Blood product transfusion received, No. (%)	38 (92.7)	12 (92.3)	26 (92.9)	.95
PRBC unit transfusion, median (IQR)	8 (5–18)	12 (6–24)	6 (3.5–14.5)	.21
D, CMV reactivation				
Days of illness at reactivation, median (IQR)	-	50 (26.5–54)	-	-
SOFA score at reactivation	-	10 (7–10)	-	-
CMV copies in plasma at reactivation, median (IQR)	-	3900 (1500–5600	-	-
CMV copies in plasma (maximum), median (IQR)	-	7500 (3900–20 100)	-	-
CMV copies in drain (maximum) (n = 14), median (IQR)	-	62 500 (27 125–85 500)	-	-
CMV copies in necrotic tissue (n = 7), median (IQR)	-	29 200 (14 700–47 500)	-	-
E, Clinical outcome				
Length of stay, median (IQR), d				
ICU	29 (20–54)	53 (27–68)	28 (18–39)	.06
Hospital	40 (28–69)	60 (29–72)	38.5 (24.5–56)	.228
Mortality, No. (%)				
At 10 wk of illness with pancreatitis	20 (48.7)	7 (53.8)	13 (46.4)	.65
At ICU discharge	24 (58.5)	9 (69.2)	15 (53.6)	.34
At hospital discharge	24 (58.5)	9 (69.2)	15 (53.6)	.34

Data are presented as median (interquartile range)/number (%), compared by Mann-Whitney *U* test/chi-square test or Fisher exact test, respectively. *P* < .05 was considered significant.

Abbreviations: APACHE II, Acute Physiology and Chronic Health Evaluation score; BSI, bloodstream infection; CMV, cytomegalovirus; CT, computed tomography; ERCP, endoscopic retrograde cholangiopancreatography; ICU, intensive care unit; IQR, interquartile range; MV, mechanical ventilation; PRBC, packed red blood cells; RRT, renal replacement therapy; SOFA, Sequential Organ Failure Assessment score.

On the day of enrollment in the study, the median (IQR) number of days of illness was 28 (14–35), the median (IQR) SOFA score was 7 (6–9), and the median (IQR) Computed Tomography Severity Index (CTSI) of pancreatitis was 10 (6–10). During ICU stay, 80% required mechanical ventilation, 93% vasopressor, and 46% renal replacement therapy (RRT). There were 24 (58%) patients who had bacteremia, and 8 (19%) also had candidemia. All except 2 required intra-abdominal drain as determined by the treating team. Thirty (73%) patients had culture-positive intra-abdominal infection (IAI), including 22% fungal infection (Table 1). Common bacterial organisms were *Escherichia coli* (19.5%), *Enterobacter cloacae* (17.1%), and *Enterococcus* species (12.2%). Among fungal infections, *Candida auris* was the most prevalent (19.5%), followed by *Candida tropicalis* (2.4%).

### CMV Reactivation

**Figure 2.**
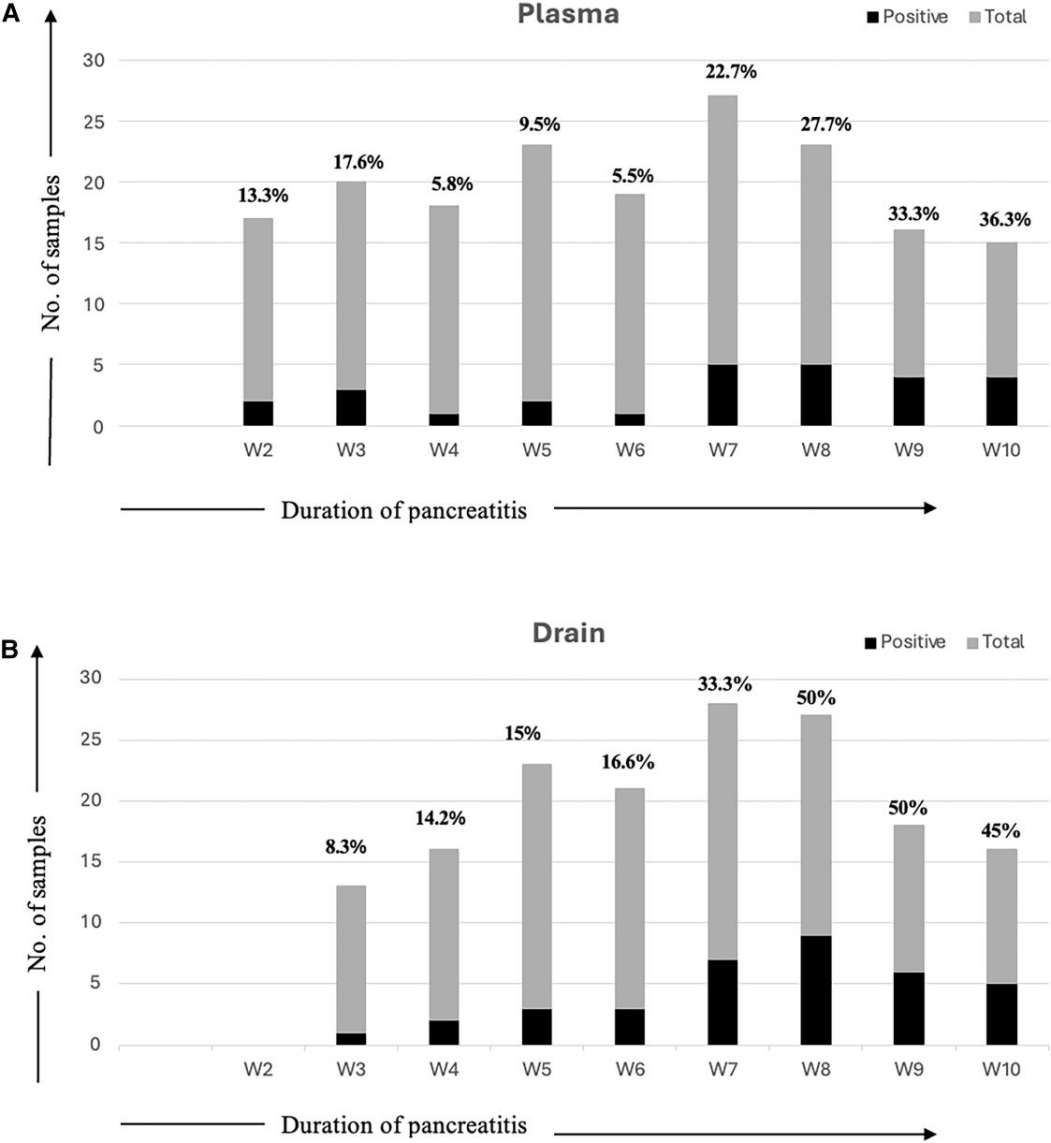
*A,* Percentage of CMV reactivation in the total analyzed plasma samples at different time points of illness. *B,* Percent positivity of raised CMV copies in the total analyzed drain samples at different time points of illness. At each time point, it is not necessarily that patients are the same, as each enrolled patients had different duration of illness at their ICU admission with varied length of ICU stay. Abbreviations: CMV, cytomegalovirus; ICU, intensive care unit.

During the study period, a total of 284 samples (plasma 149, drain content 125, and necrotic tissue 10), with the average (SD) number of plasma samples per patient being 4 (2–4) and the average (SD) number of abdominal content samples being 3 (2–4.8), were analyzed for CMV copies. CMV reactivation was detected in the plasma of 13 patients (31.7%); among them, 11 patients also had raised CMV copies in the drain content. However, there were 3 patients in the study cohort of 41 patients who had raised CMV copies in the drain content without CMV reactivation in the plasma, totaling 14 (35.8%) patients who had raised CMV copies in abdominal drain content (Table 1, Figure 2).

There were no differences in demographic or clinical characteristics at baseline in the CMV reactivation or nonreactivation groups. However, the CMV reactivation group was sicker in terms of septic shock and had a higher incidence of bloodstream infections (bacteremia as well as candidemia) and intra-abdominal infections, in comparison with the non–CMV reactivation group (Table 1).

**Figure 3.**
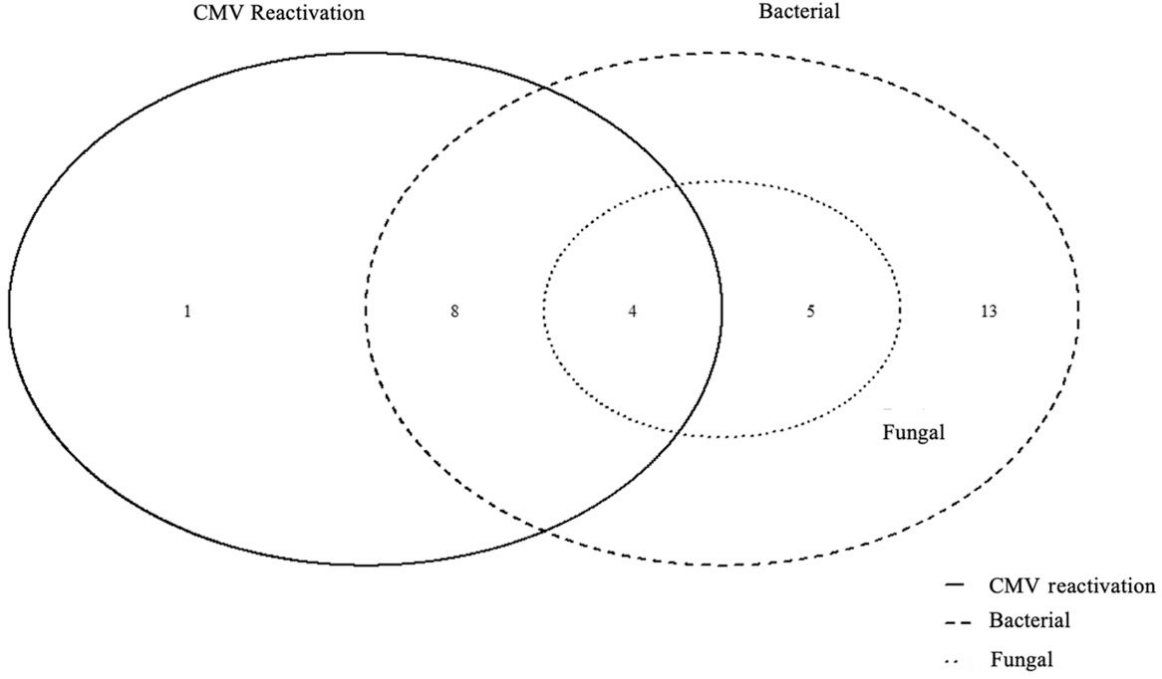
Venn diagram of concurrent intra-abdominal infections in patients with CMV reactivation (n = 13). Abbreviation: CMV, cytomegalovirus.

In the CMV reactivation group, the median (IQR) day of illness was 50 (30–52) days; these individuals had a higher SOFA score of 10 (7–10) at the time of reactivation. The average viral load was 3900 (1500–5600) copies/mL in plasma at the time of reactivation; this value rose further maximally to 7500 (3900–20 100) copies/cc in follow-up plasma samples (Table 1). Reactivation was more prevalent during the second month of illness (Figure 2; Supplementary Figure 1). Among individuals with reactivation, intra-abdominal bacterial infection was present in 92.3% and fungal in 30.7% of patients, in comparison with the nonreactivation group (64.3% and 17.8%, respectively), but the difference was statistically nonsignificant (Table 1). A Venn diagram of intra-abdominal infections showed that almost all patients with fungal and CMV reactivation had concurrent bacterial infection (Figure 3).

For patients (n = 14) in whom drain samples had raised CMV copies, the median (IQR) of the maximal copies was 62 500 (27 125–85 500) copies/mL during the study period. For patients who had CMV copies in necrotic samples (n = 7), the values were higher in tissue samples (29 200 [14 700–47 500] copies/mL) compared with concurrent drain (10 000 [4135–31 250] copies/mL) and plasma (1500 [0–12 955] copies/mL) samples from the same patients (*P* = .48).

The study cohort had a limited number of patients and did not justify the multivariate binary logistic regression analysis; an alternative method, classification and regression tree (CART) analysis, was used to test the factors and cutoff values of the variables to discriminate CMV reactivation in study patients. The CART analysis found that the ANP patients with >6 weeks of ICU stays, need for vasopressor for >2 weeks, mechanical ventilation for >3 weeks, and SOFA score >6.5 at admission were risk factors for CMV reactivation (Supplementary Figure 2).

### Clinical Outcome

At the 10th week of pancreatitis, mortality rates were found to be 53.8% in the reactivation group compared with 46.4% in the nonreactivation group (*P* = .65). However, mortality in the ICU and hospital discharge were further increased to 69.2% for the reactivation group and 53.6% for the nonreactivation group (*P* = .34). The median duration of ICU stay was longer for the reactivation group, at 60 days, compared with 38.5 days for the nonreactivation group (*P* = .06) (Table 1).

Four patients had concurrent intra-abdominal bacterial as well as fungal infections along with CMV reactivation, indicating a potentially high-risk group in which mortality was seen in 75% of patients at ICU discharge. Abdominal complications and bleeding (intra-abdominal) were present in 30% and 25% (*P* = .69), while intestinal perforation occurred in 8% and 28% (*P* = .13) of patients in the reactivation and nonreactivation groups, respectively.

**Figure 4.**
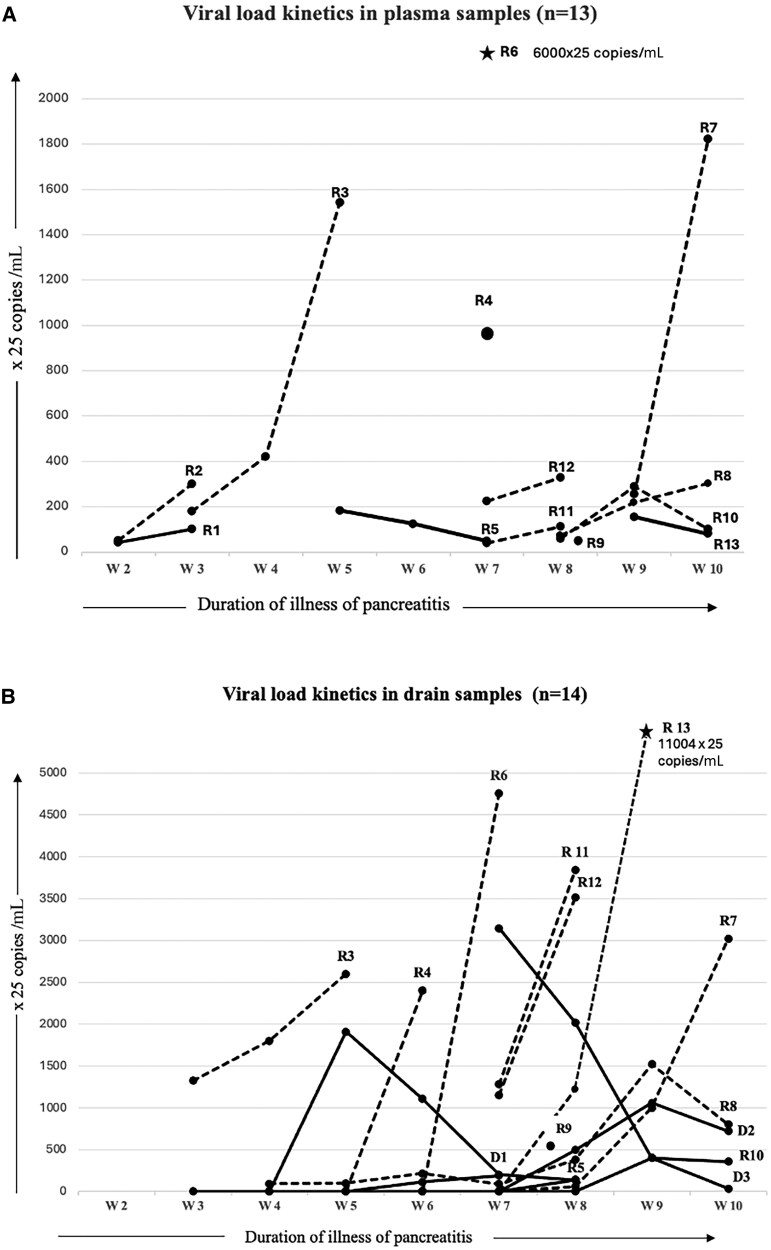
Viral load kinetics (*A*) in plasma samples of patients with CMV reactivation and (*B* ) in abdominal drain samples of patients who showed raised CMV copies. R1 to R13 numbers were assigned only to depict inclusion timing in relation to the duration of illness among patients with CMV reactivation, while D1 to D3 are those patients in whom drain samples showed raised CMV copies without raised CMV copies in the plasma (not considered CMV reactivation). Nonsurvivors are represented as broken lines (**─ ─**), while survivors are represented as solid lines (**──**); extreme high values are represented by an asterisk (*). Abbreviation: CMV, cytomegalovirus.

The viral load kinetics after the reactivation revealed higher median copies in nonsurvivors, both in plasma and drain content. Most of the nonsurvivor patients had a trend of increasing viral load in comparison with survivors (Figure 4*A* and *B*). The median (IQR) CMV copies in plasma at reactivation were higher in nonsurvivors (4462 [1500–6346] copies/mL) than in survivors (2700 [1389–4075] copies/mL; *P* = .39), which further rose to 8180 (7500–38 500) and 3200 (2250–4075) copies/mL, respectively, in follow-up samples (*P* = .01). Similarly, the maximum CMV copies in drain samples were higher in nonsurvivors (81 675 [63 750–101 750] copies/mL) compared with survivors (17 250 [6077–41 952] copies/mL; *P* = .02).

## DISCUSSION

The present study found that CMV reactivation occurred in about one-third of the ANP patients during their clinical course, with longer ICU stays and higher mortality, however nonsignificant, in comparison with ANP patients who did not have CMV reactivation. The finding of this study about the prevalence of CMV reactivation in ANP patients is comparable to those of previous studies in specific cohorts like sepsis and ARDS [3, 6], as well as nonspecific cohorts of ICU patients [22]. Different studies have used various thresholds to define CMV reactivation. Walton et al. set a threshold of 200 copies/mL in sepsis patients [3], while Ong et al. used 100 IU/mL in ARDS patients [6], both lower than the threshold in our study (1000 copies/mL). We considered a higher threshold value, thinking it would be more relevant clinically. In fact, a recent pooled analysis of prospective studies among sepsis patients by Imlay et al. demonstrated higher odds ratios for worse clinical outcomes if cutoff values were >1000 instead of >100 copies/mL [23].

The average duration of illness of ANP at the time of CMV reactivation was 6–8 weeks; however, previous studies have reported a time frame of 2–3 weeks in the general ICU population [2, 22]. This time difference of reactivation could be explained by our cohort describing time in relation to onset of illness, not from the day of ICU admission; also, in cases of severe acute pancreatitis, infection-related complication usually occurs after the third to fourth week onwards [20]. Overall, our study cohort was relatively younger (median age, 35 years) and mostly without known comorbidities. The severity of illness, including the SOFA score at ICU admission in our study cohort, was comparable to those reported in previous studies [4]. However, we observed that at the time of CMV reactivation, ANP patients had worsened organ dysfunction, with the median (IQR) SOFA score changing from 7 (6–8) to 10 (7–10).

The precise pathophysiological mechanisms underlying CMV reactivation in acutely ill pancreatitis patients remain to be fully elucidated; however, it is hypothesized that the acute inflammatory response associated with pancreatitis, compounded by superimposed sepsis, may trigger CMV reactivation via the release of pro-inflammatory cytokines such as TNF-alpha and IL-1 beta. These cytokines activate transcription factors that facilitate CMV reactivation [24]. Additionally, Venet et al. reported that septic patients exhibit immune dysfunction, characterized by reduced Th1 cell activity, increased IL-10 production, and decreased natural killer (NK) cell counts, all of which contribute to impaired interferon production [10]. This phenomenon was clinically demonstrated by Walton et al., who highlighted that reactivation of latent herpes viruses is a common occurrence in prolonged sepsis, with frequencies comparable to those observed in transplant patients receiving immunosuppressive therapy, underscoring the development of an immunosuppressive state [3]. Nonetheless, in addition to NK cells, the immunity produced by T cells plays a crucial role in the prevention of CMV reactivation. It has been demonstrated that functional CMV-specific CD8+ T lymphocyte response at ICU admission protects against CMV reactivation in critically ill nonimmunosuppressed patients [25].

A meta-analysis of 71 studies encompassing 6970 patients reported an IAI incidence of 41%, predominantly caused by gram-negative bacteria (49%–93%), followed by gram-positive bacteria (27%–86%) and fungus (0%–41%) [15]. Another meta-analysis of 22 studies involving 2151 patients by Ritu et al. observed a 26.6% incidence of fungal infections in patients with necrotizing pancreatitis [17]. Additionally, Brown et al. reported that bloodstream infections (BSIs) accounted for 8.4% of extrapancreatic infectious complications [26]. We observed a relatively higher rate of infections in our cohort of patients, for both IAI (73%) and BSI (58%). This could be explained by the longer duration of illness and hospitalization before ICU admission (median [IQR] duration of illness, 25 [14–32] days) or the higher need for organ support (vasopressor 73% and mechanical ventilation 46%) at the time of ICU admission. However, the pattern of IAI pathogens was similar, predominantly gram-negative (*E. coli* and *Enterobacter cloacae*), followed by fungal species (*Candida* non-*albicans*) and gram-positive (*Enterococcus* species). In our study, the most common fungal species was *Candida auris,* both in the intra-abdominal and blood culture samples. *Candida auris*, a “superbug fungus” because of multidrug resistance and high attributable mortality, has emerged over the last decade both in developing countries and developed countries [27].

The CMV reactivation group showed a notably higher incidence of bacterial and fungal infections than the nonreactivation group, but this was not statistically significant, probably due to the smaller number of individuals in the study cohort (Table 1). Figure 3 highlights the complexity of infection patterns in patients with ANP and CMV reactivation in our study. The fact that some patients experience multiple overlapping infections suggests that a transient immunocompromised state may predispose them to multiple infections, especially the triple-overlap central group of 4 patients (Figure 3), indicating a potentially high-risk group, with mortality of 75%.

As evidence has suggested, CMV reactivation had significant associations with adverse clinical outcomes; we also observed that the CMV reactivation group patients required more organ support, in the form of mechanical ventilation, vasopressor, and RRT, in comparison with patients without CMV reactivation. These patients also had prolonged ICU stays and higher mortality, though this was not significant, probably due to the smaller number of patients in our study. However, these findings are consistent with the findings of a meta-analysis by Lachance et al., evaluating 22 studies, which reported an extended duration of mechanical ventilation (mean difference, 6.60 days), increased need for RRT, and longer ICU length of stay (mean difference, 8.18 days) [7]. This meta-analysis also showed higher ICU mortality (odds ratio [OR], 2.55; 95% CI, 1.87–3.47) and overall mortality (OR, 2.02; 95% CI, 1.60–2.56) [7]. Similarly, another meta-analysis by Li et al. found that patients with CMV reactivation had higher mortality in patients with CMV reactivation (OR, 1.72; 95% CI, 1.04–2.85; *I*^2^ = 29%; n = 664) as compared with patients without reactivation [28].

In recent trials, prophylactic or preemptive antiviral therapy for CMV reactivation did not show mortality benefit in immunocompetent ICU patients [29, 30]. However, these trials lack statistical power, precluding any recommendation in favor of or against the prophylactic or preemptive use of antiviral therapy in these ICU populations. In fact, there is a need for further studies with an adequate number of patients to evaluate when antiviral therapy will be beneficial in terms of CMV viral load threshold for initiating the drug, as well as different types of patient cohorts with proven clinical benefits.

### Limitations and Strengths of the Study

Our study has several limitations; first, because of a limited number of ANP patients, the risk factors (like demographic, clinical characteristics, and history of ethanol intake) for CMV reactivation could not be assessed. Second, immune status at the time of CMV reactivation was not studied, which could explain the reason for the virus reactivation. Third, the association with IAI also could not be correlated statistically. Fourth, the inference for the impact of CMV reactivation on the outcome might be just a causal relationship as the sample size was not sufficient to find a significant association. However, a strength of our study is that this is the first time reporting the prevalence of CMV reactivation in ANP patients. We also analyzed CMV copies both in plasma and in abdominal drain content at multiple time intervals. These findings could lead future studies to explore the benefit of antiviral therapies among patients with CMV reactivation, with a possible impact on improved clinical outcomes among ANP patients with high morbidity and mortality.

## CONCLUSIONS

Our study revealed that CMV reactivation occurred in up to one-third of ANP patients during their clinical course of ICU management. We observed a more challenging clinical trajectory among ANP patients who had CMV reactivation with higher and persistent copies in their clinical samples; however, with the current study design (no adjustment for confounders) and limited sample size, it was not possible to conclude that CMV reactivation significantly affected or did not affect the clinical outcomes. These findings highlight the importance of investigating CMV reactivation in ANP patients in a larger sample size, which could prognosticate patients’ clinical course, and presently, the impact of treating CMV reactivation on clinical outcome is still unexplored.

## Supplementary Material

ofaf438_Supplementary_Data

## Supplementary Data

Supplementary materials are available at *Open Forum Infectious Diseases* online. Consisting of data provided by the authors to benefit the reader, the posted materials are not copyedited and are the sole responsibility of the authors, so questions or comments should be addressed to the corresponding author.
