# Supplementary figures and images for: Cytomegalovirus Reactivation in Critically Ill Patients With Acute Necrotizing Pancreatitis
Source: Open Forum Infect Dis. 2025 Jul 23;12(8):ofaf438. doi: 10.1093/ofid/ofaf438 (PMC12363391; doi:10.1093/ofid/ofaf438)

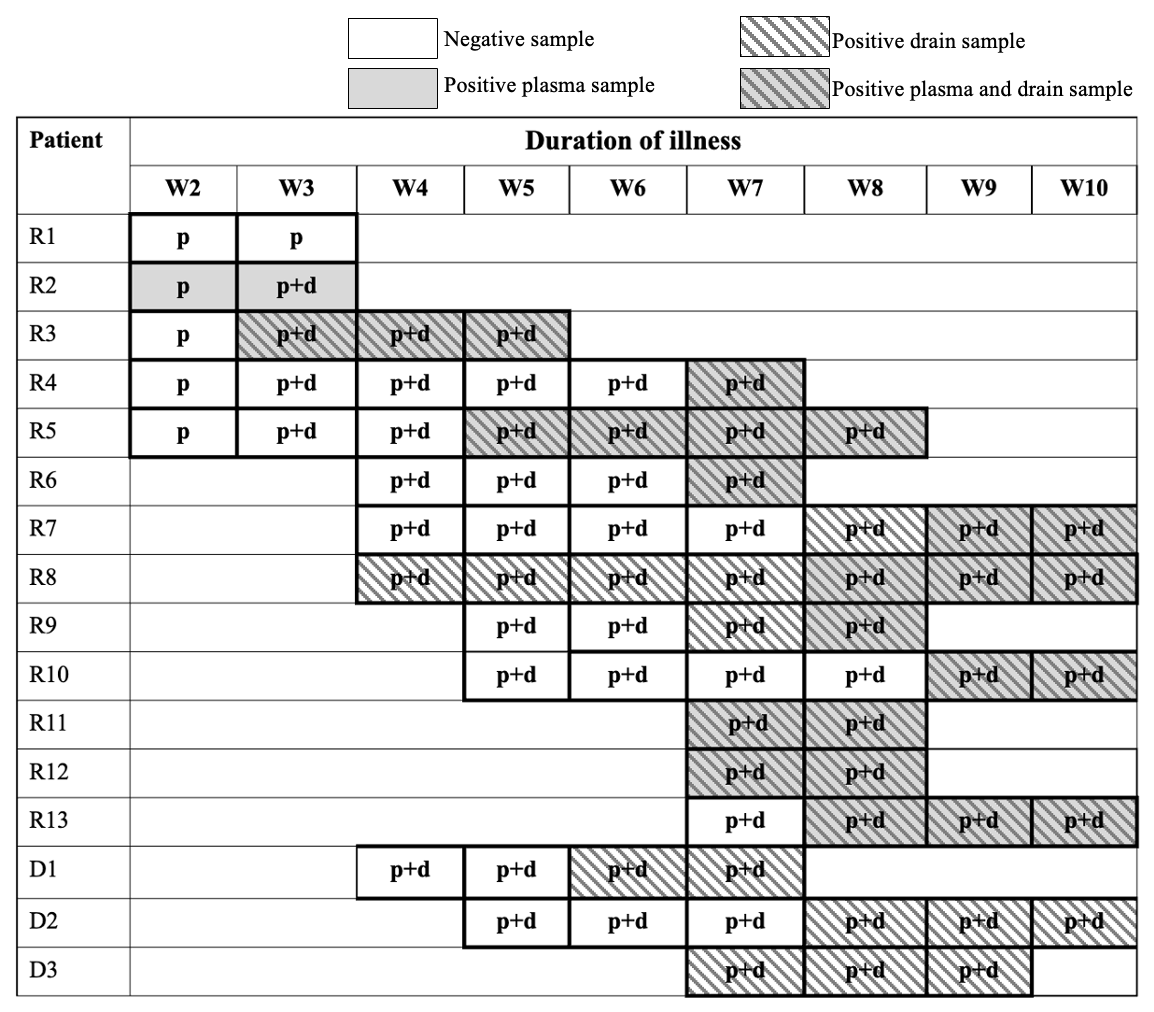

Supplement: ofaf438_Supplementary_Data [file ofaf438_supplementary_data.zip › Suppl_Figure_1.tif]

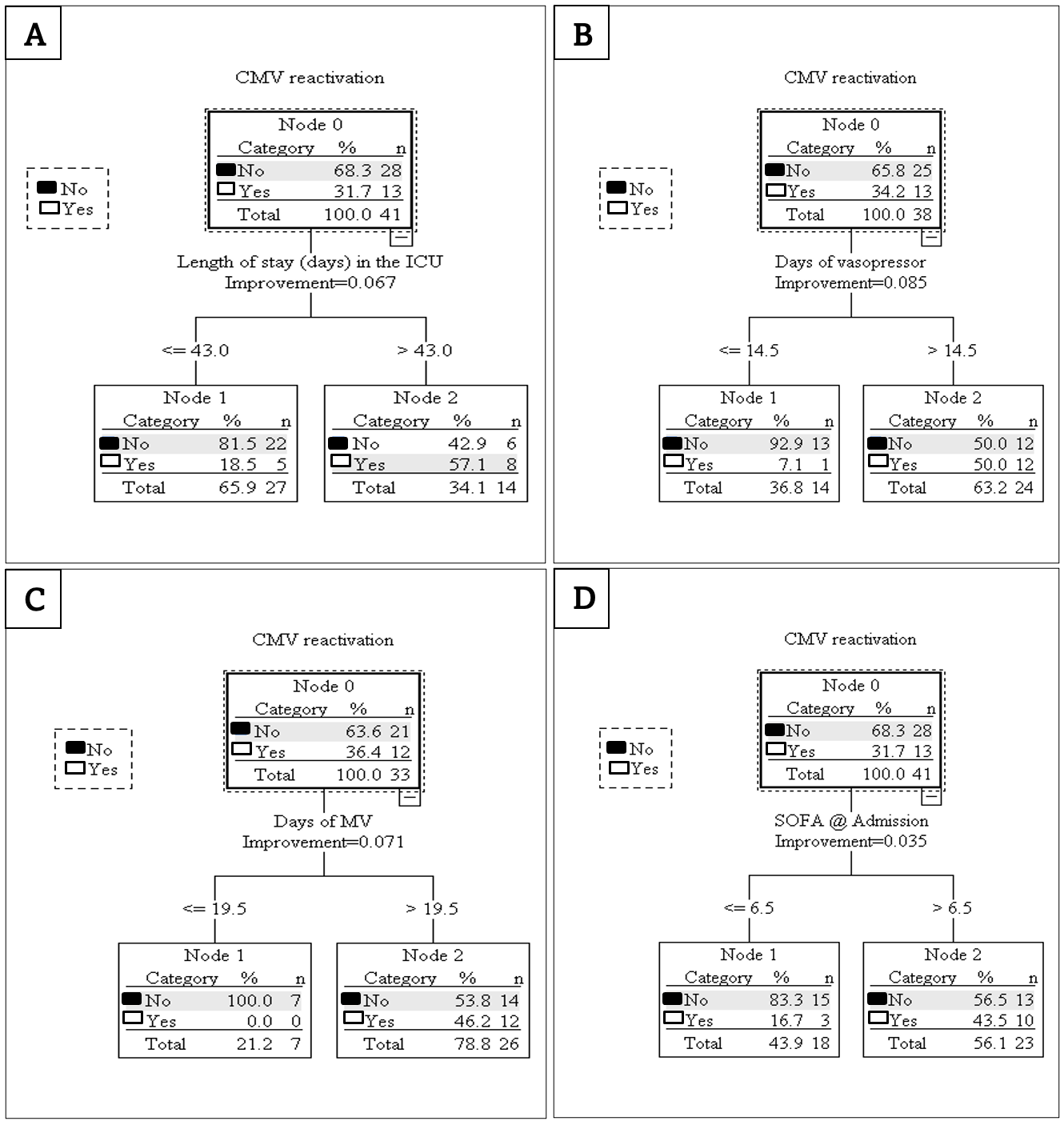

Supplement: ofaf438_Supplementary_Data [file ofaf438_supplementary_data.zip › Suppl_Figure_2.tif]
